# Nutrigenomics of Obesity: Integrating Genomics, Epigenetics, and Diet–Microbiome Interactions for Precision Nutrition

**DOI:** 10.3390/life15111658

**Published:** 2025-10-23

**Authors:** Anam Farzand, Mohd Adzim Khalili Rohin, Sana Javaid Awan, Abdul Momin Rizwan Ahmad, Hiba Akram, Talha Saleem, Muhammad Mudassar Imran

**Affiliations:** 1 Faculty of Health Sciences, School of Nutrition and Dietetics, Universiti Sultan Zainal Abidin (UNISZA), Kuala Terengganu 21300, Malaysia; si4217@putra.unisza.edu.my (A.F.); mohdadzim@unisza.edu.my (M.A.K.R.); 2 Institute of Molecular Biology and Biotechnology (IMBB), The University of Lahore, Lahore 54000, Pakistan; sana.javaidawan@yahoo.com; 3 Department of Human Nutrition and Dietetics, NUST School of Health Sciences, National University of Sciences & Technology (NUST), Sector H-12, Islamabad 44000, Pakistan; 4 Department of Health Sciences, University of York, York YO10 5NH, UK; 5 Shalamar School of Allied Health Sciences, Shalamar Medical & Dental College, Lahore 54812, Pakistan; hiba.akram1997@gmail.com; 6 Department of Medical Laboratory Technology, Superior University Lahore, Lahore 54000, Pakistan; talha.saleem@superior.edu.pk; 7 Department of Public Health and Policy, The University of Law, London EC1Y 8HQ, UK; mudassarimran878@gmail.com

**Keywords:** obesity genomics, nutrigenetics and nutrigenomics: multi-omics integration, gut microbiome diversity, GWAS (Genome-Wide Association Studies), gene–diet interactions, precision nutrition, nutrigenomics

## Abstract

Obesity is a highly complex, multifactorial disease influenced by dynamic interactions among genetic, epigenetic, environmental, and behavioral determinants that explicitly position genetics as the core. While advances in multi-omic integration have revolutionized our understanding of adiposity pathways, translation into personalized clinical nutrition remains a critical challenge. This review systematically consolidates emerging insights into the molecular and nutrigenomic architecture of obesity by integrating data from large-scale GWAS, functional epigenomics, nutrigenetic interactions, and microbiome-mediated metabolic programming. The primary aim is to systematically organize and synthesize recent genetic and genomic findings in obesity, while also highlighting how these discoveries can be contextualized within precision nutrition frameworks. A comprehensive literature search was conducted across PubMed, Scopus, and Web of Science up to July 2024 using MeSH terms, nutrigenomic-specific queries, and multi-omics filters. Eligible studies were classified into five domains: monogenic obesity, polygenic GWAS findings, epigenomic regulation, nutrigenomic signatures, and gut microbiome contributions. Over 127 candidate genes and 253 QTLs have been implicated in obesity susceptibility. Monogenic variants (e.g., *LEP*, *LEPR*, *MC4R*, *POMC*, *PCSK1*) explain rare, early-onset phenotypes, while *FTO* (polygenic) and *MC4R* (monogenic mutations as well as common polygenic variants) represent major loci across populations. Epigenetic mechanisms, dietary composition, physical activity, and microbial diversity significantly recalibrate obesity trajectories. Integration of genomics, functional epigenomics, precision nutrigenomics, and microbiome science presents transformative opportunities for personalized obesity interventions. However, translation into evidence-based clinical nutrition remains limited, emphasizing the need for functional validation, cross-ancestry mapping, and AI-driven precision frameworks. Specifically, this review systematically identifies and integrates evidence from genomics, epigenomics, nutrigenomics, and microbiome studies published between 2000 and 2024, applying structured inclusion/exclusion criteria and narrative synthesis to highlight translational pathways for precision nutrition.

## 1. Introduction

### 1.1. Global Epidemiological Landscape

Obesity is a highly prevalent, multifactorial, and biologically complex disease characterized by deregulated energy balance, adipose tissue dysfunction, and impaired metabolic homeostasis. Interconnected genomic, epigenomic, nutrigenomic, environmental, and behavioral determinants shape it [1]. Recent global estimates indicate that over 2.5 billion adults worldwide are overweight or obese, and obesity prevalence has nearly tripled since 1990, according to the Global Burden of Disease Report [2]. In the United States, the National Health and Nutrition Examination (NHANES) reports an alarming 42.4% adult obesity prevalence, with 9.2% of adults meeting criteria for class III obesity (BMI ≥ 40 kg/m^2^) [3]. Middle Eastern, Australian, and Asian regions have also shown a two- to threefold surge in obesity incidence over the last two decades, primarily due to rapid urbanization, ultra-processed dietary patterns, and sedentary lifestyles [4]. While obesity is commonly defined by a BMI ≥ 30 kg/m^2^, reliance on BMI alone underestimates visceral adiposity-driven cardiometabolic risks. Recent advances in genome-wide association studies (GWAS), functional epigenomics, microbiome profiling, and nutrigenomic modeling have revolutionized our understanding of energy homeostasis, fat distribution, and metabolic programming [5].

**Figure 1:**
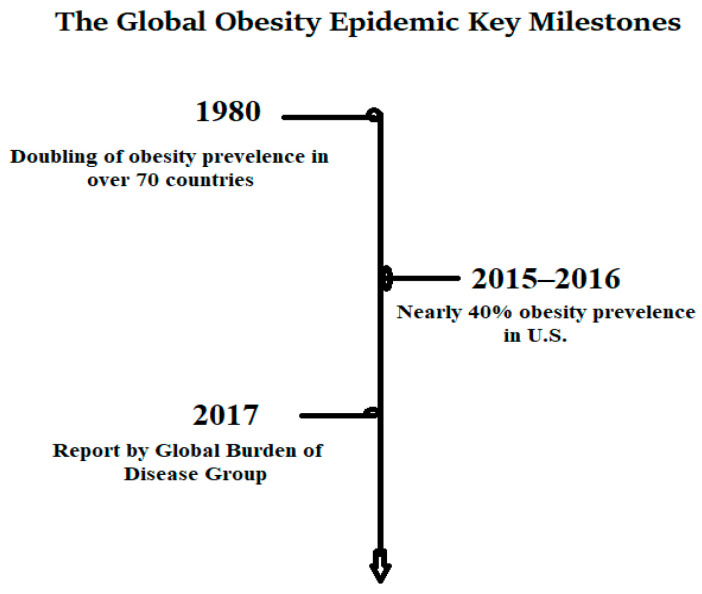
Global Obesity Epidemic.

Figure 1 shows the trends in global obesity prevalence from 1990 to 2023, highlighting a rapid acceleration in low- and middle-income countries due to urbanization, dietary transitions, and sedentary lifestyles.

However, critical knowledge gaps persist:

- Most genetic discoveries lack functional annotation, limiting clinical translation;
- Population-specific obesity loci remain underexplored, especially in Asian and low-income cohorts;
- The interaction between genetics, diet, and gut microbiota composition requires deeper integrative analysis.

Nutrigenomics is the study of how nutrients influence gene expression and metabolic pathways; it offers a transformative framework for obesity management. It focuses on precision-based dietary interventions tailored to individual genetic backgrounds, integrating nutrigenetic risk profiling, transcriptomic adaptations, and microbiome-driven metabolic regulation.

Yet, clinical translation remains limited due to fragmented datasets, inconsistent methodologies, and a lack of cross-ancestry studies.

### 1.2. Gene-Environment Framing

The genetic composition that governs energy balance, lifestyle, and perinatally imprinted behavior is responsible for excessive fat storage and obesity. In this regard, environmental factors (such as unhealthy eating and exercise habits), fetal programming, and even assortative mating may contribute to the obesity pandemic by creating a genetic predisposition. Adverse maternal nutrition and perinatal environmental exposures induce epigenetic reprogramming during fetal development, increasing lifelong obesity susceptibility [6]. Living a physically active lifestyle is linked to a 40% decrease in the genetic predisposition to common Obesity, according to a prospective study conducted on 20,000 men and women from EPIC-NORFLOK to determine the extent to which genetic susceptibility may be attenuated by such a lifestyle [7]. Estimates of heritability for obesity range from 40% to 70%, indicating a significant genetic component.

### 1.3. Objective

The objective of this review is to systematically consolidate and interpret evidence on the molecular genetics of human obesity, spanning monogenic syndromes, polygenic susceptibility loci, and gene–environment interactions. We further contextualize how epigenetic regulation, nutrigenomic responses, and microbiome dynamics influence and modulate these genetic foundations, with emphasis on translational relevance for obesity management.

Specifically, we aim to:

1. Summarize key mechanisms underlying obesity susceptibility across monogenic, polygenic, and nutrigenetic frameworks.
2. Explore diet–gene–microbiome interactions shaping adiposity and metabolic plasticity;
3. Highlight translational strategies for precision nutrition and individualized obesity prevention.

By combining multi-omics integration, machine learning–based predictive analytics, and personalized intervention frameworks, this review aims to bridge the gap between discovery science and clinical application in precision nutrition.

## 2. Methodology

### 2.1. Literature Search Strategy

Before manuscript drafting, we designed and implemented a comprehensive and systematic literature search across PubMed (National Center for Biotechnology Information, Bethesda, MD, USA), Scopus (Elsevier, Amsterdam, The Netherlands), and Web of Science (Elsevier, Amsterdam, The Netherlands) databases to identify studies relevant to nutrigenomics, obesity genetics, and multi-omics metabolic regulation. 

### 2.2. Databases and Keywords

The search covered studies published between January 2000 and July 2024, with a specific focus on identifying the most recent five-year literature (2020–2024). Using a combination of Medical Subject Headings (MeSH), nutrigenomic-specific keywords, and Boolean operators to refine queries. Search strings included: “Obesity genomics”, “nutrigenomics”, “multi-omics integration”, “GWAS”, “epigenomic regulation”, “gene–diet interactions”, “microbiome metabolic pathways”, and “precision nutrition”. This structured approach ensured that the evidence base directly guided the organization and interpretation of this review, rather than being applied retrospectively.

### 2.3. Eligibility Criteria

Studies were considered for inclusion if they:

- Investigated genetic, epigenetic, nutrigenomic, or microbiome-driven factors influencing obesity susceptibility;
- Reported findings from large-scale GWAS, epigenomic profiling, nutrigenetic studies, or gut microbiome analyses;
- Explored diet–gene interactions or nutrient-responsive molecular pathways;
- Published in peer-reviewed, high-quality journals.

Exclusion Criteria:

- Non-original works are excluded unless they provide meta-analytical insights;
- Animal-only studies without human translational relevance;
- Grey literature and preprints are not peer-reviewed.

### 2.4. Study Selection Process

The selection was conducted in two independent stages:

- Title & Abstract Screening: Two independent reviewers screened all retrieved studies based on predefined inclusion criteria;
- Full-Text Evaluation: Articles meeting the initial screening were reviewed in detail to extract key findings related to obesity-associated genes, nutrigenomic pathways, and microbiome-modulated mechanisms.

### 2.5. Data Extraction and Synthesis

Data from eligible studies were extracted into a structured framework, including:

- Study design, sample size, and demographic details;
- Genetic loci, gene variants, and pathway-level findings;
- Nutrigenomic outcomes, microbiome diversity indices, and epigenomic signatures.
- Reported diet–gene–microbiome interactions.

A narrative synthesis framework was applied, integrating cross-study findings using:

- Functional pathway mapping to identify molecular convergence;
- Gene-environment response profiling to analyze nutrigenomic interactions;
- Cross-ancestry meta-analysis insights where available.

## 3. Genetic Basis of Obesity

Genetic factors influence metabolic set points, shaping how individuals regulate body weight and respond to obesity interventions [8]. Hundreds of genes across multiple biological pathways govern energy balance, lipid storage, and adiposity regulation, thereby influencing weight gain and weight-loss responses [9].

**Figure 2:**
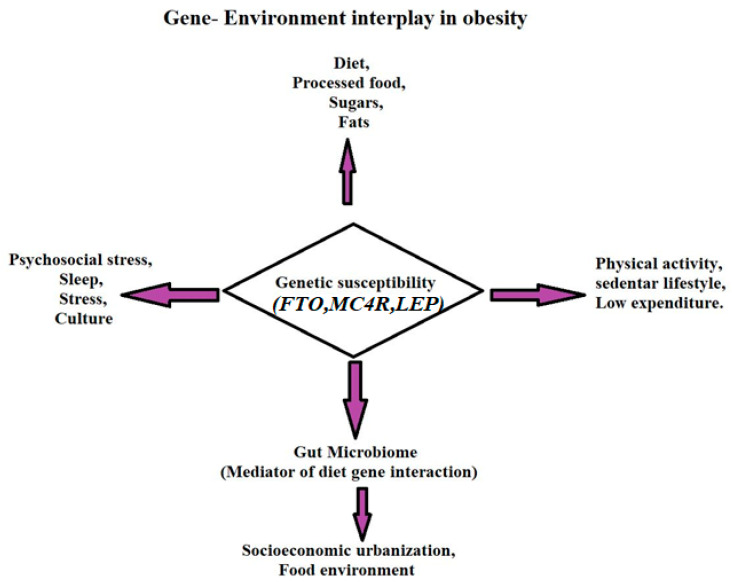
Genetic and Environmental Factors in Obesity.

As shown in Figure 2, obesity arises from a multifactorial interplay of genetics and environment. Rather than acting independently, environmental exposures, including diet quality, physical activity, socioeconomic conditions, and psychosocial stress, interact with genetic predispositions (e.g., *FTO*, *MC4R*, *LEP*) to modulate obesity risk. This interaction framework better reflects the dynamic nature of gene–environment pathways underlying adiposity.

Animal models have been instrumental in mapping key obesity-related pathways, with emerging human studies confirming several candidate genes. However, these candidate genes explain only a small fraction of heritability, suggesting obesity is primarily a polygenic trait involving complex interactions among numerous loci [10]. Even with strong genetic predisposition, environmental factors—such as diet quality, physical activity, and socioeconomic context substantially modify obesity risk. Familial aggregation studies reveal that individuals with two obese parents have a markedly higher likelihood of developing obesity compared to those with lean parents. Historical evidence dating back to the 1960s supports a strong genetic contribution to body weight regulation. Adoption and twin studies consistently demonstrate that body weight is more strongly correlated with biological parents than adoptive ones, reinforcing the heritable basis of obesity [11].

**Figure 3:**
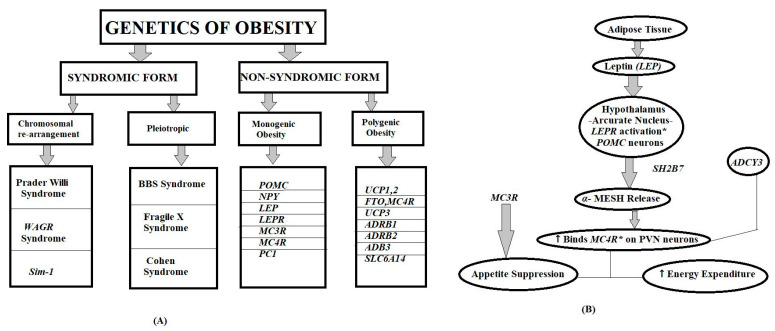
Molecular Genetics of Human Obesity (Leptin–Melanocortin Pathway). Leptin (*LEP*), secreted by adipose tissue, binds to its receptor *(LEPR*) in the arcuate nucleus of the hypothalamus, leading to activation (*) of *POMC* neurons and subsequent release of α-MSH. α-MSH binds to *MC4R* receptors on paraventricular nucleus (PVN) neurons, promoting appetite suppression and increased energy expenditure, and arrows (↑) indicate the direction of signal transduction or biological effect.

Figure 3A illustrates the classification of genetic forms of obesity, categorized into syndromic and non-syndromic types. Syndromic obesity includes chromosomal rearrangement–related and pleiotropic disorders such as Prader–Willi syndrome, Bardet–Biedl syndrome, and Cohen syndrome. Non-syndromic obesity is further subdivided into monogenic and polygenic forms. Genes implicated in monogenic obesity include *POMC*, *LEP*, *LEPR*, *MC3R*, and *MC4R*, while polygenic obesity involves variants in genes such as *FTO*, *UCP1-3*, *MC4R*, *ADRB1-3*, and *SLC6A14*. Figure 3B Schematic representation of the leptin–melanocortin signaling pathway. 

### 3.1. Monogenic Obesity Syndromes

#### 3.1.1. Discovery and Historical Context

Insights from rodent models have driven the discovery of several monogenic obesity syndromes in humans [12]. Monogenic obesity arises from single-gene mutations that disrupt energy homeostasis and typically follow Mendelian inheritance patterns, leading to severe, early-onset obesity. The idea that a circulating factor may mediate energy homeostasis was solidified with the subsequent development of multiple murine genetic models of Obesity and their investigation in parabiosis experiments by Coleman [13]. However, the molecular elements of an energy balance regulation network were not put together until the 1990s, when the exact molecular foundation for the agouti, ob/ob, db/db, and fat/fat mice appeared [14]. Several new monogenic diseases that cause Obesity in humans have surfaced in recent years [15].

#### 3.1.2. Key Genes Implicated and Clinical Outcomes

Mutations in key genes, *POMC*, *MC4R*, *LEP*, *LEPR*, and *PCSK1*, have been strongly associated with severe, early-onset obesity as shown in Table 1 [16]. Leptin, encoded by the *LEP* gene, regulates appetite, energy expenditure, and adipose metabolism. Circulating leptin levels are positively correlated with body fat percentage, showing elevated concentrations in obesity and reduced levels in cachexia or anorexia. Recent studies have shown that leptin also modulates neuroendocrine pathways, influencing the secretion of *FSH*, *LH*, *ACTH*, cortisol, and growth hormone [17]. The leptin gene on chromosome 7 (7q31.3) is mutated in congenital leptin deficiency. This gene codes for a protein of 167 amino acids and consists of three exons and two introns [18]. Recent pharmacogenomic studies have further highlighted therapeutic opportunities for monogenic obesity, particularly through *MC4R* agonists and leptin replacement strategies [19].

Furthermore, it has recently been demonstrated that leptin affects the control of FSH, LH, ACTH, cortisol, and GH concentrations in addition to its effects on food intake and energy expenditure [21]. The *LEP* gene on chromosome 7 (7q31.3) is mutated in congenital leptin deficiency. This gene codes for a protein of 167 amino acids and consists of three exons and two introns [22]. Three members of a consanguineous family were identified with a leptin receptor mutation.

**Figure 4:**
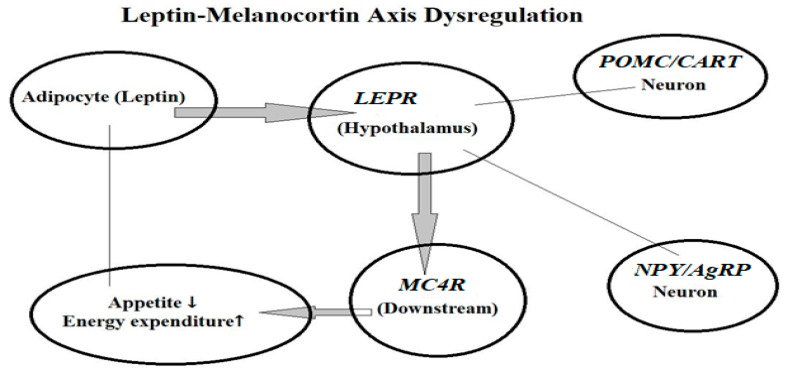
Genetic and Molecular Factors in Obesity: Leptin–Melanocortin Axis Dysregulation: Schematic representation of the leptin–melanocortin signaling pathway and its dysregulation in obesity. Leptin, secreted from adipocytes, binds to leptin receptors (*LEPR*) in the hypothalamus. This interaction activates pro-opiomelanocortin (*POMC*) and cocaine- and amphetamine-regulated transcript (CART) neurons, while simultaneously inhibiting neuropeptide Y (*NPY*) and agouti-related peptide (*AgRP*) neurons. The cascade subsequently activates the downstream melanocortin-4 receptor (*MC4R*), leading to reduced appetite (↓) and increased energy expenditure (↑).

Figure 4 visualizes the regulatory role of leptin, *LEPR*, *MC4R*, and downstream neuroendocrine pathways in controlling appetite, energy expenditure, and fat storage.

A mutation that truncates the receptor before the transmembrane domain was found to be homozygous in affected individuals. Despite having a normal birth weight, leptin receptor-deficient individuals gained weight quickly in the first few months of life, displaying significant hyperphagia and hostile behavior when food was refused [23]. Food intake and energy balance are significantly impacted by *MC4R*, which is also expressed in the hypothalamus [24]. Severe Obesity, severe hyperinsulinemia, increased lean body mass, and linear growth of the five known melanocortin receptors are clinical characteristics of mutant carriers; *MC4R* has been most closely associated with regulating energy balance in rats [25,26]. A unique role for the MC4-R in the control of energy balance is defined by the obesity syndrome that results from its absence, which is very similar to the Agouti disease. This suggests that the underlying cause of the agouti obesity syndrome is abnormal antagonism of the *MC4-R*. An alternate explanation for the finding that MC4-R signaling directly affects food and metabolism is that deletion of the *MC4-R* gene may cause a malfunction in the development of the hypothalamus, which would lead to hyperphagia and hypometabolism. For instance, Obesity is linked to abnormalities in the ventromedial hypothalamus’s function in humans and rats [27]. *MC4R* mutant individuals exhibit higher lean mass and increased fat mass, which is not observed in leptin insufficiency.

**Figure 5:**
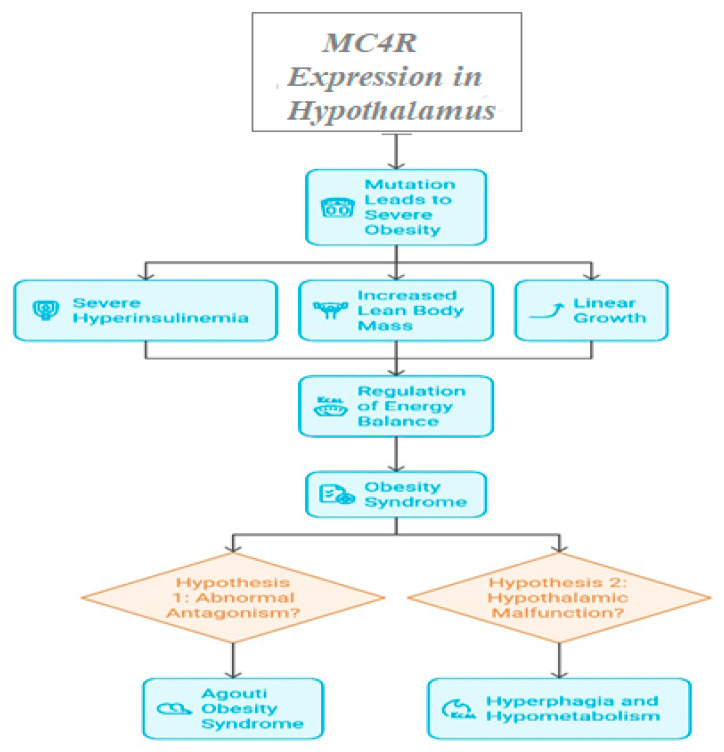
*MC4R* Expression and Energy Homeostasis.

Figure 5 depicts *MC4R*-associated variants and their role in hypothalamic regulation of feeding behavior, lean mass accrual, and energy balance.

### 3.2. Polygenic Obesity and GWAS Findings

Unless explicitly stated otherwise, all figures and tables in this section refer to human genetic association data (e.g., polymorphisms) rather than experimental knockdown models. Obesity is increasingly recognized as a neuroendocrine-driven condition, arising from complex gene–environment interactions. Recent large-scale molecular studies demonstrate that most obesity risk arises from the cumulative effects of numerous common variants [28].

**Figure 6:**
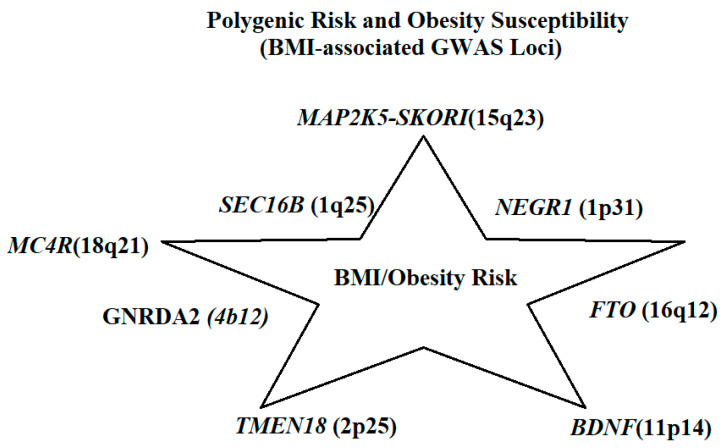
Polygenic Risk and Obesity Susceptibility.

Figure 6 provides a schematic summary of the most consistently replicated BMI-associated GWAS loci, including *FTO*, *MC4R*, *TMEM18*, *NEGR1*, *SEC16B*, *BDNF*, *GNPDA2*, and *MAP2K5–SKOR1*. These loci, also detailed in Table 2, represent the most robust polygenic contributors to obesity risk across populations. While *FTO* is exclusively polygenic, *MC4R* represents a unique case: rare mutations cause monogenic obesity, whereas common polymorphisms act as major polygenic risk factors. Polygenic variation refers to small-effect alleles across multiple loci, collectively influencing quantitative traits such as BMI and fat distribution. Individuals carrying multiple risk alleles have a significantly higher probability of developing obesity compared to those with fewer or no variants [29]. Within *MC4R*, two well-characterized polymorphisms, Ile251Leu and Val103Ile, have protective associations, demonstrating reduced obesity risk among carriers [30]. Recent GWAS identified rs17782313, located 188 kb downstream of *MC4R*, as one of the strongest BMI-associated variants, highlighting its central role in obesity susceptibility. A comprehensive overview of both major polygenic loci identified through GWAS and key appetite-regulation genes implicated in obesity susceptibility is provided in Table 2. This integrated summary highlights the convergence between common variants influencing adiposity and monogenic pathways affecting appetite regulation.

A multi-cohort GWAS meta-analysis involving 16,800+ European participants confirmed the variant’s strong association with BMI. After *FTO*, this SNP showed the second-highest genome-wide significance, likely mediated by *MC4R* transcriptional regulation. Findings were validated across >60,000 adults, 6000 children, and multiple family-based cohorts, underscoring robust reproducibility. The average difference in BMI for each copy of the rs17782313 C-allele was around 0.22 kg/m^2^ (760 g). Overweight and Obesity odds ratios rose by 8 and 12% with one copy of the C-allele, respectively; no discernible gender differences were seen. Fat mass had a disproportionate impact on weight [46]. Cross-ancestry comparisons enhance understanding of shared biological mechanisms and enable fine-mapping of obesity-susceptibility loci with higher precision [47]. The latest edition of the “Human Obesity Gene Map” provides an excellent summary of this; it includes 127 candidate genes, of which slightly less than 20% have been replicated by five or more studies, 244 knockout or transgenic animal models, 50 loci linked to Mendelian syndromes relevant to human Obesity, and 11 single gene mutations. Out of 61 genome-wide linkage scans, 253 quantitative trait loci (QTL) for various obesity-related traits have been identified. Just over 20% of them have several studies to support them [48]. Across diverse ancestries, *FTO* and *MC4R* consistently emerge as the most replicated obesity loci, demonstrating cross-population robustness. First discovered in Europeans, single-nucleotide polymorphisms (SNPs) in *FTO* and close to *MC4R* are obesity-susceptibility loci that extensively replicate across various ancestries. According to SNP-to-SNP comparisons, individuals of East Asian and European ancestry share almost half of the loci linked to the 36-body mass index. Locus-wide investigations, however, suggest that the transferability may be considerably greater [27].

**Figure 7:**
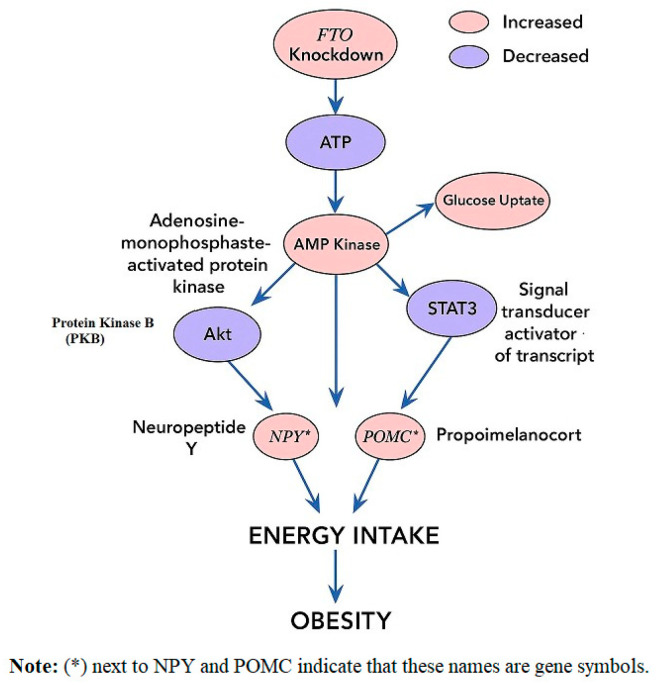
Proposed Mechanistic Role of *FTO* in Adiposity.

Figure 7 Integrates structural, transcriptional, and functional evidence explaining how *FTO* polymorphisms, as identified in GWAS and functional studies, modulate appetite regulation and fat deposition. This figure reflects the effects of genetic variants rather than *FTO* knockdown experiments.

## 4. Epigenetic Modifications in Obesity

Beyond genetic variation, epigenetic mechanisms play a pivotal role in regulating obesity risk by modulating gene expression without altering the DNA sequence. Epigenetics encompasses DNA methylation, histone modifications, and non-coding RNAs that dynamically influence transcriptional activity [49].

**Figure 8:**
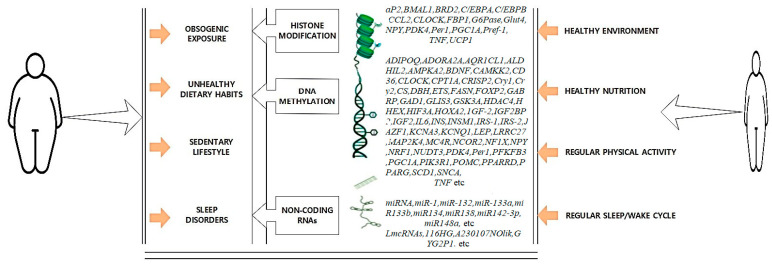
Epigenetic regulation of genes related to Obesity. An illustration of the most prevalent epigenetic modifications and targeted genes researched in the context of obesogenic versus healthy lifestyle choices.

Figure 8 highlights the influence of DNA methylation, histone modifications, and non-coding RNAs on transcriptional control of adipogenesis and energy homeostasis.

DNA methylation and histone modifications act as tissue-specific regulatory markers, influencing energy metabolism and adipocyte differentiation. Genomic imprinting further regulates obesity-associated loci by controlling parent-of-origin–specific allele expression. In humans, there are two primary clusters of genomic imprinting: a region at 11p15 that contains many imprinted genes, including *H19*, *KCNQ1*, *CDKN1C*, *PHLDA2*, and *KVLQT1* (maternally expressed) and *IGF2*, *INS*, and KCNQ10T1 (*LIT1*) (paternally expressed). At least seven imprinted genes, including *MKRN3*, *MAGEL2*, *NDN*, *SNURF–SNRPN* (paternally expressed), and *UBE3A*, *ATP10A* (maternally expressed), are found in the second cluster at 15q11–q12. Aberrant imprinting failures disrupt normal growth and differentiation pathways, predisposing individuals to early-onset obesity. For instance, Prader–Willi syndrome arises from 15q11–q13 deletions or uniparental disomy, leading to hyperphagia and severe obesity. A condition marked by hyperphagia (induced by satiety center malfunction) and severe (often fatal) early-onset obesity [50]. Recent meta-analyses demonstrate that *LEP* and *ADIPOQ* promoter methylation strongly associate with obesity and weight-loss response. Moreover, miRNAs such as miR-33 and miR-103 modulate lipid metabolism, while long non-coding RNAs like HOTAIR influence adipogenic differentiation. AI-driven epigenomic profiling has recently uncovered novel methylation signatures linked to metabolic plasticity in obesity, underscoring the translational potential of big-data approaches [51].

## 5. Genetic Mechanisms Underlying Obesity

### 5.1. Appetite Regulation Genes

A complicated condition, Obesity is influenced by several hereditary and environmental variables. It has also recently been proposed that uncommon genetic variations with potent effects may be the cause of Obesity. Monogenic Obesity is linked to gene mutations related to the hypothalamic leptin-melanocortin signaling pathway. *LEP*, *LEPR*, *POMC*, *PCSK1*, *MC4R*, *MC3R*, *SH2B1*, *NTRK2*, *MRAP2*, and *TUB* are some of these genes. New genes linked to Obesity, as shown in Table 2, that are involved in the melanocortin pathway or hypothalamic development have recently been discovered. These genes include *ADCY3*, *MYT1L*, *POU3F2*, *GRPR*, and *LRP2* [20].

Cholecystokinin and other short-term hormonal, psychological, and neurological cues from the gastrointestinal system regulate eating behavior. On the other hand, different cues, including the newly identified hormone ghrelin, could trigger eating. Long-term energy storage is indicated by circulating nutrients, insulin, leptin, and other hormones. Low energy stores cause adipose tissue to produce less leptin, which lowers circulating leptin concentrations. This results in a decrease in α-melanocyte-stimulating hormone (α-MSH), cocaine, and amphetamine-regulated transcript (CART), as well as an increase in hypothalamic neurotransmitters that strongly increase food intake, such as neuropeptide Y (*NPY*), galanin, and agouti-related protein (*AGRP*). A 28-amino acid peptide called ghrelin was extracted from the stomach of rats. The human stomach mucosa’s endocrine cells are its primary source. However, it was also discovered in several other tissues, including the kidney, placenta, ovary, testis, pituitary, hypothalamus, pancreas, lung, and immune cells [43].

### 5.2. Fat Metabolism and Storage Genes

*VAT* and *SAT* were used to measure the gene expression of the *VLDL* receptor (*VLDLR*), lipoprotein lipase (*LPL*), acylation stimulating protein (ASP), *LDL* receptor-related protein 1 (*LRP1*), and fatty acid binding protein 4 (*FABP4*) in 28 morbidly obese patients with Type 2 Diabetes Mellitus (T2DM) or high IR, 10 morbidly obese patients with low IR, 10 obese patients with low IR, and 12 lean, healthy controls.

**Figure 9:**
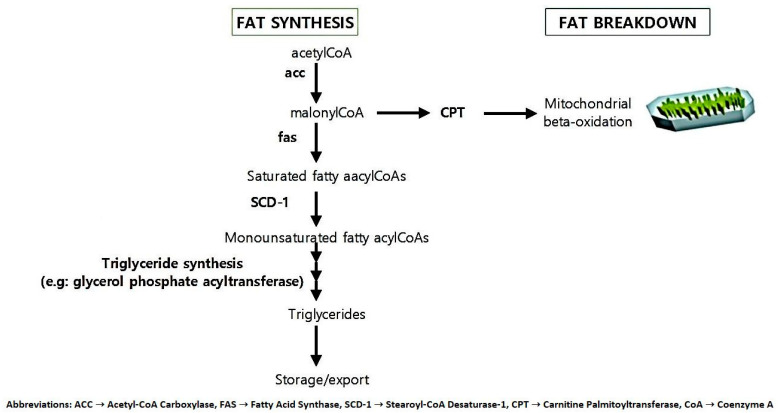
Fat metabolism and storage disease underlying Obesity: Adipose Tissue Lipid Metabolism Pathways.

Figure 9 illustrates the comparative dysregulation of lipid storage, lipolysis, and fatty acid mobilization pathways in obese versus lean phenotypes. The schematic highlights differential gene expression (e.g., upregulation of *NPY1R* and *CES1*; downregulation of *CIDEA*, *APOE*, and *ABCA*) observed in human adipose tissues. These differences represent an altered regulatory balance between storage and mobilization processes, rather than direct molecular dysregulation. Lipid storage begins with the introduction of FFA into the adipocyte. Lipoprotein lipase (*LPL*) lipolyzes the triglycerides carried in triglyceride-rich lipoproteins (*TRL*), such as chylomicrons and VLDL, to FFA, subsequently absorbed by the adipocyte. *LPL*, attached to glycosaminoglycans at the luminal side of the capillary endothelium, catalyzes the rate-limiting step for triglyceride catabolism or FFA accumulation in peripheral tissues, including adipose tissue. The main enzyme that controls the entrance and esterification of FFA in adipose tissue is LPL [52]. Differential gene expression in subcutaneous abdominal adipose tissue samples from people with lean and obese phenotypes was examined and validated using microarray and RT-PCR analysis [53]. The findings include the following: When comparing obese and lean people, NPY1R and CES1 were upregulated, whereas several genes and transcripts implicated in lipolysis, including *AKAP1*, *PRKAR2B*, *Gi*, and *CIDEA*, were downregulated. Similarly, transcripts linked to lipoprotein and cholesterol metabolism were expressed differently in obese compared to lean patients, with *VLDLR* rising and *APOE* and *ABCA* falling [31].

### 5.3. Energy Expenditure Genes

**Figure 10:**
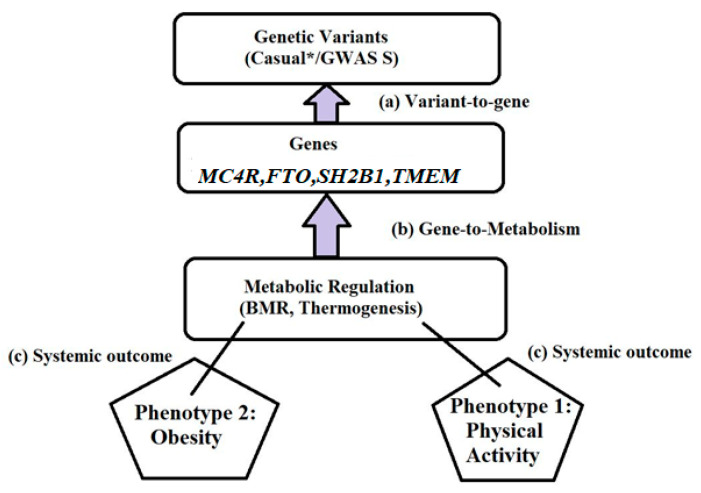
Energy Expenditure Genes Network: In this figure (* “Causal” indicates genetic variants that have a direct biological effect on the phenotype, distinguishing them from variants that are only correlated through genome-wide association studies (GWAS)).

It is well acknowledged that one of the variables contributing to the genesis of Obesity is individual variability in energy expenditure. Energy expenditure is a complicated phenomenon frequently examined concerning its many elements. These elements include energy expenditure, the thermic impact of meals, resting and basal metabolic rates, and activity-related energy expenses. The additive genetic impact, also known as heritability, and the genotype-environment interaction effect are the two genetic effects that are often considered [54,55]. We explicitly visualized these causal links in Figure 10 by arranging panels (a–c) sequentially with directional flow, thus clarifying the upstream–downstream relationships across genetic, mitochondrial, and systemic levels of energy regulation.

**Figure 11:**
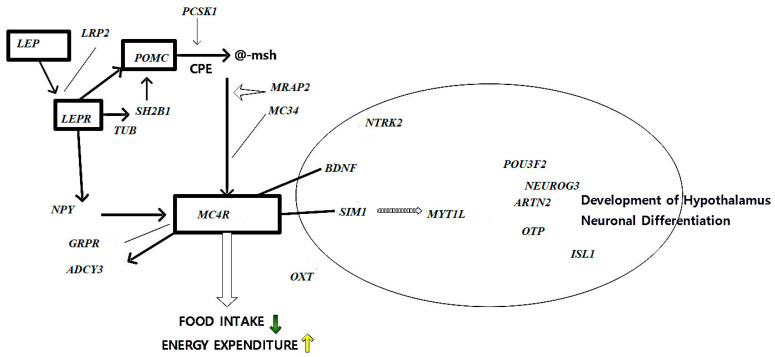
Gene’s energy expenditure: Schematic representation of the leptin–melanocortin signaling pathway, Leptin (*LEP*) binds to the leptin receptor (*LEPR*), activating downstream components such as *SH2B1* and *TUB*, which in turn stimulate pro-opiomelanocortin (*POMC*) expression. *POMC* is processed by *PCSK1* and *CPE* to produce α-MSH, which activates the melanocortin 4 receptor (*MC4R*). *MC4R* signaling regulates food intake and energy expenditure through interactions with *GRPR*, *ADCY3*, and neuronal factors such as *SIM1*, *MYT1L*, and *BDNF* via *NTRK2*. The right panel illustrates transcription factors (*POU3F2*, *NEUROG3*, *ARTN2*, *OTP*, *ISL1*) involved in hypothalamic development and neuronal differentiation. Note: Solid arrows represent direct activation or signaling relationships, while dashed arrows (e.g., between *SIM1* and *MYT1L*) indicate indirect or transcriptional regulation. The downward green arrow denotes decreased food intake, and the upward yellow arrow indicates increased energy expenditure.

To clarify the mechanistic continuum, Figure 10 is now arranged in a stepwise manner: panel (a) illustrates thermogenesis-related genes as upstream drivers, panel (b) depicts mitochondrial regulators of basal metabolic rate as intermediate nodes, and panel (c) integrates downstream systemic variability in energy expenditure. This sequential arrangement highlights how upstream genomic variants propagate through mitochondrial activity to shape whole-body metabolic outcomes. Even after adjusting for the well-established concomitants of energy expenditure, variations in human energy consumption can be partially attributed to the impact of the genotype, as shown in Figure 11. Evidence from classical twin studies, adoption cohorts, and family aggregation analyses consistently demonstrates that genetic factors explain approximately 35–45% of interindividual variance in resting metabolic rate, the thermic effect of food, and energy cost of low-to-moderate intensity exercise [56]. More recent genome-wide association studies (e.g., *FTO*, *MC4R*, *UCP2*, *PPARG* loci) and polygenic risk score analyses further corroborate this heritability estimate, confirming that both traditional quantitative genetics and molecular approaches converge on a ~40% genetic contribution [57].

A significant genetic effect of habitual physical activity has also been reported in Figure 11. The existence of a genotype-environment interaction has also been investigated. Thus, in response to chronic overfeeding and negative energy balance, changes in the components of energy expenditure exhibit significant identical twin pair resemblance. Nutrient partitioning is emerging as a major determinant of individual differences in metabolic rate responses to overfeeding or adverse energy balance conditions. Taken as a whole, these observations consistently support the hypothesis that heredity plays a significant role in the various components of energy expenditure in humans [58].

#### 5.3.1. Gene Influencing Basal Metabolic Rate

According to quantitative genetics research, a large portion of the phenotypic variance in BMR may be ascribed to an additive genetic component. This is mainly highlighted by several genes with minor effects that code for protein structural polymorphism. Nevertheless, environmental influences and non-additive genetic factors, which most likely work by modifying gene expression, account for an additional 40% of BMR variance [59].

#### 5.3.2. Role of Physical Activity-Related Genes in Obesity Risk

The World Health Organization identified Obesity as the most significant risk to Westernized countries’ health in 2000. Nearly 40% of adults in the US suffer from Obesity, which causes more than 400,000 deaths annually. Even though Obesity may be controlled with medication, diet, and exercise, rates have been rising. For instance, in the EPIC-Norfolk study of 20,000 adults, physically active individuals with high-risk *FTO* alleles had a 40% lower obesity prevalence compared to inactive carriers. Similar findings were replicated in Amish and European cohorts, underscoring that exercise attenuates polygenic obesity risk [60].

According to earlier research, physical activity and genetic risks are inversely correlated, meaning that increased physical activity might help reduce a greater genetic risk for Obesity, as shown in Table 3. Daily step counts from fitness tracking devices were used to quantify activity monitoring. A polygenic risk score (PRS) from a comprehensive genome-wide association study (GWAS) of BMI was used to measure genetic risk. We calculated the average number of steps per day required to compensate for the hereditary risk of elevated body mass index [61].

Over the past several decades, the incidence of Obesity has dramatically increased, and higher levels of physical activity have been linked to lower levels of body fat and metabolic risk. Genetic variables are also crucial in developing Obesity, according to genetic epidemiology research, as shown in Table 4 [64].

## 6. Gene–Environment Interaction in Obesity

Obesity represents a global health burden, predisposing individuals to cardiometabolic and endocrine disorders. The rapid escalation of obesity prevalence reflects gene–environment interactions, where genetic predisposition interacts with lifestyle, diet, and socioeconomic determinants [67]. Over 60% of individuals in the United States are thought to be overweight or obese. Similar increases have been seen in the proportion of kids and teenagers who are overweight (i.e., ≥95th percentile) or at risk for being overweight (i.e., ≥85th percentile) [68].

### Interaction Between Genetic Predispositions and Environmental Factors

The strong impact of the environment on an individual’s susceptibility to Obesity is demonstrated by migration studies conducted on immigrants arriving in America using data from the National Health Interview Survey [69]. Other comparisons of high-risk ethnic groups living in contrasting environmental settings [70], and randomized controlled trials of weight loss interventions [71]. This evidence supports a model where genetic susceptibility amplifies the effects of obesogenic environments, accelerating disease onset. The impact of genes on Obesity has been the subject of much contemporary research. Numerous genes have been connected to Obesity by scientists, indicating that genes play a significant role in developing this illness. Numerous genes found by GWA scans, including *MC4R* (melanocortin-4 receptor) and *FTO* (fat mass and obesity-associated), have been firmly linked to the risk of Obesity in a variety of populations [72]. But when it comes to Obesity, we still do not fully understand how genes impact how our bodies react to dietary or energy changes. Nutrigenomic studies, as shown in Table 5 reveal that genetic variants regulate dietary responsiveness, affecting lipid storage, appetite control, and metabolic flexibility [73]. Numerous metabolic problems, including insulin resistance, hyperglycemia, and dyslipidemia, are often linked to Obesity. Additionally, Obesity is linked to an increased risk of coronary heart disease, type 2 diabetes, asthma, sleep apnea, hypertension, some types of cancer, and other mortality [74].

Lipid and lipoprotein phenotypes have been used in most research on the relationship between human genes and food. According to several assessments of these investigations, the response to diet is modulated by genetic diversity in the LDL receptor gene, multiple apolipoproteins, and LDL subclass phenotypes [76]. Physical activity improves insulin sensitivity, lipid metabolism, and cardiometabolic outcomes across populations. However, exercise response is highly genotype-dependent, with specific variants modulating fat oxidation and energy expenditure [77]. In obese people, the relationship between genes and nutrition is a complicated research topic. It entails comprehending how hereditary variables impact how each person reacts to various food regimens, including metabolic wellness and weight control.

**Figure 12:**
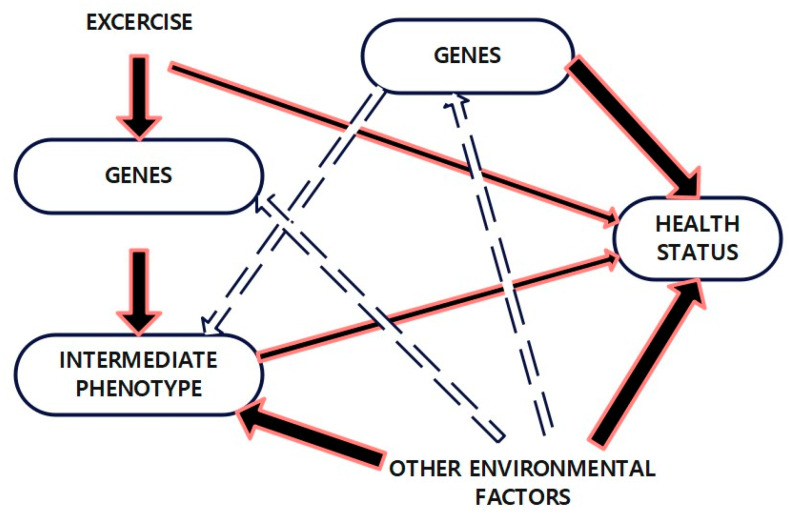
Genetic environmental factors: This diagram depicts the multifactorial relationships that contribute to overall health status. Exercise influences gene expression and intermediate phenotypes (e.g., metabolic rate, cardiovascular function), which in turn affect health outcomes. Genetic factors interact bidirectionally with intermediate phenotypes, modulating the effects of both exercise and environmental exposures. Note: Solid red arrows: Indicate direct effects or influences (e.g., exercise directly affecting gene expression or health status). Thick black arrows: Represent strong causal pathways or major determinants of health (e.g., genetic and environmental effects on health status). Dashed arrows: Depict indirect or feedback interactions (e.g., how genes and intermediate phenotypes mutually influence each other or how environmental factors affect gene–phenotype relationships). Bidirectional arrows: Show reciprocal interactions where both components influence each other (e.g., gene–phenotype or gene–environment feedback loops).

Figure 12 demonstrates how lifestyle interventions, including diet and physical activity, can attenuate or amplify genetically mediated obesity risk.

Increased physical activity may mitigate the negative consequences of *FTO* gene variations, according to 2008 research on Amish people. The findings imply that physical exercise might mitigate the elevated risk of Obesity caused by genetic vulnerability to *FTO* mutations. These results highlight how important physical exercise is to public health initiatives to fight Obesity, especially in those genetically predisposed to the condition [78].

## 7. Role of Gut Microbiota in Obesity

Numerous microorganisms, including bacterial, fungal, and protozoal, comprise our microbiota and are found in the human body [79]. Managing gut microbiota has emerged as a novel approach to treating Obesity [80]. Through several processes, gut microbes may impact weight growth and fat deposition. Short-chain fatty acids, especially butyrate, enhance *GLP-1* and *PYY* secretion, improving satiety and glucose control. Conversely, increased lipopolysaccharide-producing Gram-negative bacteria drive metabolic endotoxemia, promoting chronic inflammation and obesity. One reason is the large intestine’s microbes’ capacity to ferment ordinarily indigestible food ingredients to release energy. The host indirectly obtains this energy through the absorption of short-chain fatty acids generated by microbes [81]. Studies involving obese people following diets low in carbs or diets containing various non-digestible carbohydrates have demonstrated that the species composition of the gut microbiota varies with diet composition. The genera Methano-bacterial, Lactobacillus, Bifidobacterium, and Akkermansia, as well as the family Christen senellaceae, are typically regarded as probiotics, and Obesity is frequently inversely correlated with their relative abundance [82]. However, there is conflicting data about how much the makeup of the gut microbiota varies between those who are obese and those who are not [83]. The number of bacteria that break down fiber is linked to weight reduction, whereas the presence of bacteria that break down protein and fat is linked to weight gain. Numerous variables, including genetics, influence the gut microbiome; however, the functional relationships between these factors are not as well-known due to the diversity of the microbial community [84]. Numerous complicated human illnesses and host characteristics have been connected to the microbiome. Human intervention trials published in recent years confirm that modulation of gut microbiota composition through dietary fibers or probiotics directly influences adiposity outcomes [85].

**Figure 13:**
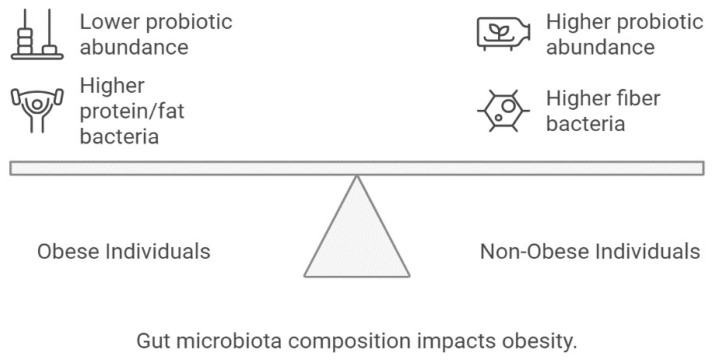
Gut microbiota composition impacts Obesity.

Figure 13 visualizes the taxonomic shifts in microbial diversity linked to differential energy extraction, metabolic flexibility, and inflammatory pathways.

## 8. Genetic and Clinical Implications for Future Directions

Current pharmacotherapies primarily target central appetite regulation, gut–hormonal pathways, adipose metabolism, and hepatic lipid processing. Emerging interventions—including gene therapy, microbiome modulation, anti-obesity vaccines, and next-generation drug delivery systems are under active investigation [86]. Advances in molecular genetics have accelerated gene therapy approaches aimed at correcting obesity-linked metabolic defects. These strategies aim to enhance energy expenditure, restore leptin–melanocortin signaling, and reprogram adipose metabolism. Delivering coding or non-coding gene sequences can restore homeostatic protein networks, improving long-term metabolic regulation. Many genes, including those that code for the proteins and enzymes involved in food intake, lipogenesis, lipolysis, glucose metabolism, and fat storage in adipose tissue, must work in concert and balance to maintain metabolic homeostasis. The metabolic processes are also significantly influenced by the genes that control the expression of these vital genes. Obesity may result from the over- or under-expression of one or more genes necessary for metabolic balance [87]. Respect for autonomy in the context of genetic testing and screening refers to a person’s right to make an educated, autonomous decision regarding whether or not they want to be tested and whether or not they want to be told of the specifics of the test’s results.

**Figure 14:**
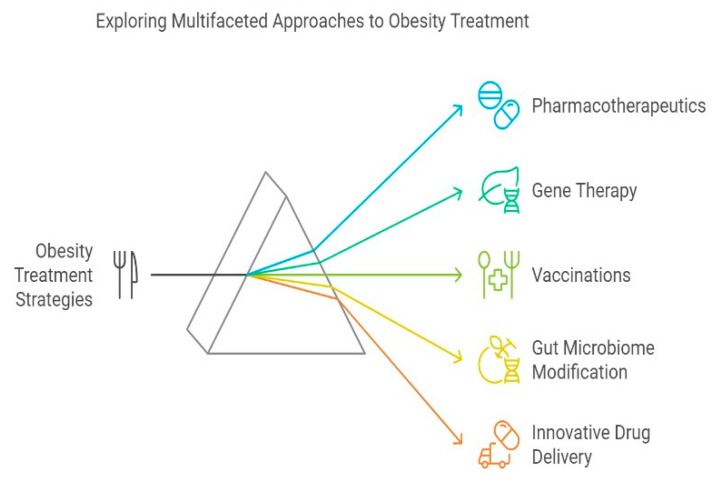
Obesity Treatment Strategies: Clinical Implications of Genetic Discoveries.

Figure 14 summarizes translational strategies, including gene therapy, microbiome modulation, anti-obesity vaccines, and personalized pharmacogenomics.

Additionally, autonomy refers to the individual’s right to be in charge of their fate, whether or not genetic information is involved, and to prevent others from interfering with significant life decisions, whether or not they are influenced by genetic information. The right to manage the future use of gene material submitted for analysis for a particular purpose is another aspect of respect for autonomy. This includes the possibility of storing the genetic material and the information derived from it for later analysis, such as in a DNA bank or registry file [88]. Health disparities in obesity stem from the interaction of genetic, environmental, and socioeconomic determinants. Precision medicine is becoming feasible through advances in genomic profiling, multi-omics integration, and machine-learning–based risk prediction. Nonbiological variables like socioeconomic position are frequently the cause of health inequalities between white people and vulnerable social groups like racial/ethnic minorities. Indeed, race is not a biological term; instead, it is a societal construct. However, from the standpoint of health inequities, it is worthwhile to investigate how genetics contributes to the manifestation of common illnesses [75]. Integrating genetic data into clinical practice remains limited, highlighting the urgent need for translational pipelines connecting genomics to treatment decisions. Implementation science might be utilized to address the obstacles and difficulties and investigate prospects and optimal approaches for incorporating genetic data into standard clinical and public health procedures [89]. Based on what is currently known about the biology and pathophysiology of the illness, the candidate gene method is a hypothesis-driven strategy. This method looks for a correlation between a characteristic of interest (like Obesity) and a variation or mutation in or close to the candidate gene. The most recent translational pipelines leverage CRISPR-Cas9 genome editing and AI-based dietary prediction models to personalize obesity therapy [90].

Genome-wide linkage studies examine whether certain chromosomal regions are correlated with a disease or characteristic over generations and are dependent on the relatedness of research participants. In genetics research, a genome-wide association study is used to identify correlations between certain diseases or features and a large number (usually hundreds of thousands) of distinct genetic variants, most often single-nucleotide polymorphisms [91]. Numerous genes are connected to Obesity, according to genome-wide association studies (GWASs). These discoveries provide insight into various unique prospective weight-management therapies, such as genome editing. Given its capacity to alter DNA or alter gene expression in eukaryotic cells, the state-of-the-art technology known as clustered regularly interspaced palindromic repeats (CRISPR)/CRISPR-associated protein (Cas) surely helps us comprehend the genetic mechanisms underlying Obesity. It may prove to be a helpful treatment tool [92].

According to the few molecular marker studies released thus far, several genes are probably connected to human Obesity. This research has been given additional impetus by recent advancements in animal genetics, transfection systems, transgenic animal models, recombinant DNA technologies used in positional cloning, and techniques to discover loci contributing to quantitative characteristics [93].

**Figure 15:**
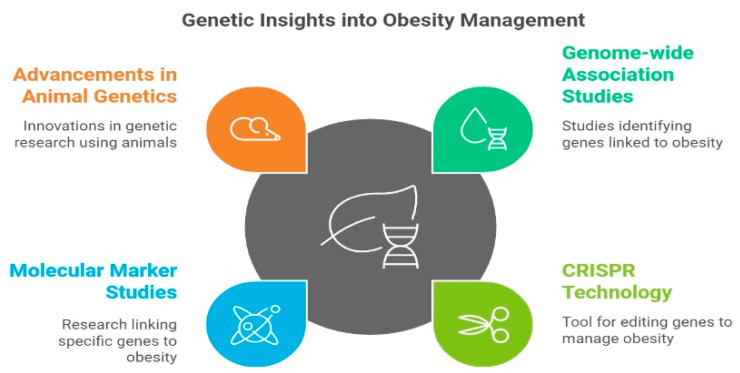
Genetic Insights of Obesity Management.

Figure 15 shows a comprehensive framework summarizing the integration of genetic, epigenetic, and environmental determinants of obesity.

We anticipate that the integration of various data sources will not only make it possible to identify a growing number of loci that contribute to obesity susceptibility but also deepen our understanding of the pathogenetic mechanisms underlying these variants in a field with quickly changing technologies and analytical methodologies. Ultimately, these developments will support a more thorough comprehension of Obesity as an illness and enable the customization of upcoming treatment strategies [94].

## 9. Limitations and Strengths

Undertaking genetic research for complex diseases like Obesity offers numerous advantages. Genetic technology has the potential to reveal new biological pathways and identify fresh targets for intervention in preventing, treating, or curing diseases caused by multiple factors. Furthermore, a better understanding of human genetics will enhance our knowledge of the disease, improving prevention, diagnosis, and management. Nevertheless, there are obstacles, such as the necessity for large sample sizes and the complexity of defining consistent traits. Genetic research also encounters the challenge of heterogeneity, with limited genetic findings on Obesity despite long-term studies. Even genome-wide association analyses have yielded minimal results. It is evident that genetics plays a role in Obesity, but is not the sole cause. Furthermore, translating genetic discoveries into clinical use is arduous, and ethical considerations must be carefully observed when conducting genetic research on human populations. While our systematic approach ensured methodological rigor, the exclusion of non-English and grey literature may have led to selection bias.

## 10. Conclusions and Future Genetic Perspectives in Obesity

This review consolidates the molecular genetic basis of obesity as the primary determinant, with additional modulation by epigenetic, nutrigenomic, and microbiome factors. It also systematizes the genetic architecture of obesity, consolidating recent discoveries across monogenic and polygenic loci. Building on this genetic foundation, we further integrate epigenomic, nutrigenomic, and microbiome evidence to highlight translational opportunities for precision nutrition. Obesity arises from a complex interplay of genetic predisposition, epigenetic regulation, and environmental exposures, with hundreds of loci identified through GWAS and multi-omics studies. Despite significant advances, critical gaps remain in understanding variant functionality, gene–environment dynamics, and population-specific risks.

Integrating genomic insights with epigenetic profiling, nutrigenomics, and AI-driven risk modeling will accelerate the transition from discovery to precision-based clinical applications. Leveraging these approaches will enable personalized prevention strategies, targeted therapies, and predictive modeling of obesity susceptibility.

Future research should focus on:

- Functional characterization of obesity-associated loci;
- Cross-ancestry genomic mapping to address diversity gaps;
- Integrating multi-omics datasets with machine learning for risk prediction;
- Designing personalized interventions that optimize diet, physical activity, and metabolic health.

With advances in multi-omics integration, functional genomics, and artificial intelligence, the field is moving toward a paradigm of predictive, preventive, and personalized healthcare. A deeper understanding of the molecular mechanisms driving obesity will transform its diagnosis, treatment, and long-term management.

## Figures and Tables

**Table 1:** Key Monogenic Genes Linked to Severe Early-Onset Obesity.

Gene	Encoded Protein	Primary Function	Clinical Impact	References
*LEP*	Leptin	Regulates appetite & energy balance	Congenital leptin deficiency → severe hyperphagia	[14]
*LEPR*	Leptin receptor	Mediates leptin signaling	Impaired satiety, early-onset obesity	[15]
*MC4R*	Melanocortin-4 receptor	Controls food intake & energy expenditure	Accounts for ~5% of severe early-onset obesity	[12]
*MC3R*	Melanocortin-3 receptor	Regulates energy balance	Variants associated with obesity phenotypes	[20]
*POMC*	Pro-opiomelanocortin	Precursor for α-MSH, binds MC4R	Mutations cause extreme hyperphagia & adrenal dysfunction	[17]
*PCSK1*	Prohormone convertase 1	Activates appetite-regulating peptides	Loss-of-function → defective energy regulation	[17]

**Table 2:** Key Genetic Loci and Appetite Regulation Genes Associated with Obesity Susceptibility.

Gene/Locus	Chromosomal Location	Encoded Protein/Gene Name	Biological Function	Associated Trait/Relevance	Replication/References
*FTO*	16q12	Fat mass and obesity-associated protein	Epigenetic regulation of energy balance	BMI, fat deposition	Strong, multiple populations [31,32]
*MC4R*	18q21	Melanocortin-4 receptor	Regulates appetite & energy expenditure	BMI, obesity, and appetite control	Strong, replicated across ancestries [33]
*MC3R*	20q13.2–13.3	Melanocortin-3 receptor	Regulates energy homeostasis	BMI, obesity	Moderate, Caucasian and Hispanic populations [20,34]
*TMEM18*	2p25	Transmembrane protein 18	Body fat storage	BMI	Consistent replication [35]
*NEGR1*	1p31	Neuronal growth regulator 1	Regulation of adiposity and waist circumference	BMI, waist circumference	Strong [36]
*SEC16B*	1q25	SEC16 homolog B	Visceral fat regulation	BMI, fat distribution	Consistent [37]
*BDNF*	11p14	Brain-derived neurotrophic factor	Satiety and neuronal differentiation	BMI, extreme obesity	European cohorts [38]
*GNPDA2*	4p12	Glucosamine-6-phosphate deaminase 2	Appetite modulation and obesity risk	BMI	Moderate, replicated in Europeans [39]
*MAP2K5–SKOR1*	15q23	MAP kinase pathway genes	BMI regulation	BMI	Strong in Europeans [40]
*SLC6A14*	Xq23	Solute carrier family 6, member 14	Amino acid transport, nutrient sensing	Obesity	French/Finnish cohorts [41]
*PCSK1*	5q15–q21	Proprotein convertase subtilisin/kexin type 1	Activation of appetite-regulating peptides	BMI, obesity	East Asian replication [8]
*APOA2*	1q23	Apolipoprotein A-II	Modifies lipid metabolism depending on diet	Diet–gene interaction (saturated fat)	Functional nutrigenomic evidence
*LEP*	7q31.3	Leptin	Regulates appetite & energy balance	Congenital leptin deficiency, hyperphagia	Disease-causing [16]
*LEPR*	1p31	Leptin receptor	Mediates leptin signaling	Impaired satiety, early-onset obesity	Disease-causing [20]
*POMC*	2p23.3	Pro-opiomelanocortin	Precursor for α-MSH, binds MC4R	Hyperphagia & adrenal dysfunction	Disease-causing [16]
*ADCY3*	2p23.3	Adenylate cyclase 3	Hypothalamic cAMP signaling	Severe obesity variants	Associated [20]
*ARNT2*	15q25	Aryl hydrocarbon receptor nuclear translocator 2	Hypothalamic neuronal differentiation	Developmental role	Animal models [42]
*CPE*	4q32.3	Carboxypeptidase E	Neuropeptide processing	Obesity-associated variants	[41]
*GRPR*	Xq22	Gastrin-releasing peptide receptor	Satiety regulation	Obesity variants	[20]
*ISL1*	5q11.2	ISL LIM home-box 1	POMC expression, hypothalamic neuron differentiation	Developmental role	Linkage analysis
*LRP2*	2q31.1	LDL receptor-related protein 2	Enhances leptin-induced STAT3	Obesity variants	[20]
*MYT1L*	2p25.3	Myelin transcription factor 1-like	Hypothalamic development	Obesity variants	[20]
*NPY*	7p15.3	Neuropeptide Y	Stimulates food intake	Obesity variants	[43]
*NTRK2*	9q22	BDNF receptor	Hypothalamic differentiation	Disease-causing	[20]
*OTP*	5q13.1	Orthopedic home-box	Hypothalamic development	Animal model	[20]
*OXT*	20p13	Oxytocin	Appetite regulation	Hypothalamic circuit role	[20]
*NEUROG3*	10q21.3	Neurogenin 3	Hypothalamic transcription factor	Developmental role	Animal model [42]
*POU3F2*	6q16.1	POU class 3 home-box 2	Hypothalamic transcription factor	CNV studies	[20]
*SH2B1*	16p11.2	Src homology 2 B adapter protein 1	Modulates leptin & insulin signaling	Hyperphagia, obesity	Disease-causing [44]
*SIM1*	6q16.3	Single-minded homolog 1	Hypothalamic differentiation	Disease-causing	[45]
*TUB*	11p15	Tubby transcription factor	Hypothalamic neuropeptides	Syndromic obesity	[44]

**Table 3:** Gene development and obesity complications.

Gene/Mutation	Function in the Development of Obesity Complications	References
Gene of adiponectin (variants rs1501299, rs2241766, rs266729 and rs17300539)	Marker of cardiometabolic risk	[62]
*SREBF1*	Responsible for the increased risk of coronary heart disease in patients with obstructive sleep apnea	[63]
Deletion of the long arm of chromosome 15	Prader–Willi syndrome-increased obstructive sleep apnea risk	[62]
Rs926198 variant of the gene encoding caveolin-1	Increased risk of cardiovascular disease in obesity	[62]
Genes of transcription factor TCF7L2 and *PPAR-γ2* receptor	Occurrence of type 2 diabetes mellitus in the obese	[55]
*SLC16A11* gene variants	Development of type 2 diabetes in the inhabitants of Mexico and other Latin American countries	[55]
Gene encoding the amyloid A	The size of the adipocytes increased in obese people	[62]
*PPP1R15A*, *HADHA*, *NR1P1*, *FOS*, *FOSB* and *JUN*	Co-existence of osteoporosis, colon cancer, and Obesity	[62]
Polymorphism *Ala55Val* of *UCP2* gene	Weight loss in obese patients undergoing laparoscopic adjustable gastric banding	[55]

**Table 4:** Examples of Obesity-Susceptible Loci Identified By GWAS.

Gene	Chromosomal Location	Phenotype	Population Studied	References
*FTO*	16q12	BMI; WC; Fat percentage; extreme obesity	European, African, Asian	[32]
*MC4R*	18q21	BMI; WC; extreme obesity	European: Indian Asian	[33]
*MC3R*	20q13.2–13.3	Obesity	The Caucasian population, the Hispanic population	[34]
*SLC6A14*	Xq23	Obesity	Finish, French	[41]
*POMC*	2p23.3	BMI	European	[65]
*BDNF*	11p4	BMI; extreme Obesity	European	[38]
*TMEM18*	2p25	BMI; extreme Obesity	European	[35]
*NEGR1*	1p31	BMI	European	[36]
*PCSK1*	5q15-q21	BMI	East Asian	[39]
*GNPDA2*	4p12	BMI	European	[66]
*MAP2K5*	15q23	BMI	European	[40]
*SEC16B*	1q25	BMI	European	[37]

**Table 5:** Nutrigenomic Variants and Diet–Gene Interactions.

Gene/SNP	Dietary Modifier	Metabolic Effect	Phenotypic Impact	References
*FTO* rs9939609	High protein vs. high carb	Alters IRX3/IRX5 expression	Protein-rich diets mitigate BMI risk	[75]
*APOA2* CC genotype	Saturated fat	Modifies lipid storage	High saturated fat intake an increase (↑) BMI	[16]
*PPARγ2* Pro12Ala	Dietary fats	Enhances insulin sensitivity	Improved glucose metabolism on high MUFA diets	[58]
*TCF7L2* variants	Refined carbohydrate load	Influences β-cell function	Modulates diabetes & adiposity risk	[62]

## Data Availability

No new data were created or analyzed in this study. Data sharing is not applicable to this article.

## Abbreviations

The following abbreviations are used in this manuscript:

BMI	Body Mass Index
GWAS	Genome-Wide Association Study
SNP	Single-Nucleotide Polymorphism
NGS	Next-Generation Sequencing
DNA	Deoxyribonucleic Acid
RNA	Ribonucleic Acid
mRNA	Messenger RNA
miRNA	MicroRNA
lncRNA	Long Non-Coding RNA
CpG	Cytosine–Phosphate–Guanine (dinucleotide site)
ChIP-seq	Chromatin Immunoprecipitation Sequencing
ATAC-seq	Assay for Transposase-Accessible Chromatin Sequencing
eQTL	Expression Quantitative Trait Locus
CRISPR	Clustered Regularly Interspaced Short Palindromic Repeats
*FTO*	Fat Mass and Obesity-Associated Gene
*MC4R*	Melanocortin 4 Receptor
PCOS	Polycystic Ovary Syndrome
NASH	Non-Alcoholic Steatohepatitis
T2D	Type 2 Diabetes
HDL	High-Density Lipoprotein
LDL	Low-Density Lipoprotein
GWAS-CNV	Genome-Wide Association Study–Copy Number Variant
MR	Mendelian Randomization
ACC	Acetyl-CoA Carboxylase
FAS	Fatty Acid Synthase
SCD-1	Stearoyl-CoA Desaturase-1
CPT	Carnitine Palmitoyltransferase
CoA	Coenzyme A
